# Functional mechanism and pathogenic potential of MYRF ICA domain mutations implicated in birth defects

**DOI:** 10.1038/s41598-020-57593-8

**Published:** 2020-01-21

**Authors:** Hongjoo An, Chuandong Fan, Mohamed Sharif, Dongkyeong Kim, Yannick Poitelon, Yungki Park

**Affiliations:** 1 Hunter James Kelly Research Institute, Department of Biochemistry, Jacobs School of Medicine and Biomedical Sciences, State University of New York at Buffalo, Buffalo, NY 14203 USA; 2 Department of Neuroscience and Experimental Therapeutics, Albany Medical College, Albany, NY 12208 USA

**Keywords:** Transcription factors, Development, Gene expression

## Abstract

Myrf is a membrane-bound transcription factor that plays a key role in various biological processes. The Intramolecular Chaperone Auto-processing (ICA) domain of Myrf forms a homo-trimer, which carries out the auto-cleavage of Myrf. The ICA homo-trimer-mediated auto-cleavage of Myrf is a prerequisite for its transcription factor function in the nucleus. Recent exome sequencing studies have implicated two MYRF ICA domain mutations (V679A and R695H) in a novel syndromic form of birth defects. It remains unknown whether and how the two mutations impact the transcription factor function of Myrf and, more importantly, how they are pathogenic for congenital anomalies. Here, we show that V679A and R695H cripple the ICA domain, blocking the auto-cleavage of Myrf. Consequently, Myrf-V679A and Myrf-R695H do not exhibit any transcriptional activity. Molecular modeling suggests that V679A and R695H abrogate the auto-cleavage function of the ICA homo-trimer by destabilizing its homo-trimeric assembly. We also found that the ICA homo-trimer can tolerate one copy of Myrf-V679A or Myrf-R695H for its auto-cleavage function, indicating that V679A and R695H are not dominant negatives. Thus, if V679A and R695H in a heterozygous state caused birth defects, it would be via haploinsufficiency of *MYRF*.

## Introduction

Myrf (myelin regulatory factor, previously known as *C11orf9* [the human gene] and *Mrf* [the mouse gene]) is a pleiotropic membrane-bound transcription factor^1–7^. Throughout this paper, *MYRF* and *Myrf* refer to the human and mouse genes, respectively. In the murine central nervous system (CNS), *Myrf* is specifically expressed by oligodendrocytes (OLs)^1,8^. Conditional knockout of *Myrf* in OL lineage cells led to widespread dysmyelination and severe neurological deficits^1^. Myrf is also indispensable for the life-long maintenance, plasticity, and regeneration of CNS myelin^9–11^. These studies left the impression that Myrf is a “myelin” transcription factor, and hence the name Myrf. We now know that *MYRF* is also expressed in other tissues such as stomach, lung, heart, ovary, eye, and developing gonads^12–15^. Consistently, *MYRF* coding variants have been implicated in both myelin and non-myelin diseases^13–19^, and whole-body *Myrf* knockout led to embryonic lethality in mouse independent of OL development^1^. In keeping with myelin-independent functions of Myrf, *Myrf* orthologs are found and play an important role in organisms without myelin^2,3,6,7^.

**Figure 1:**
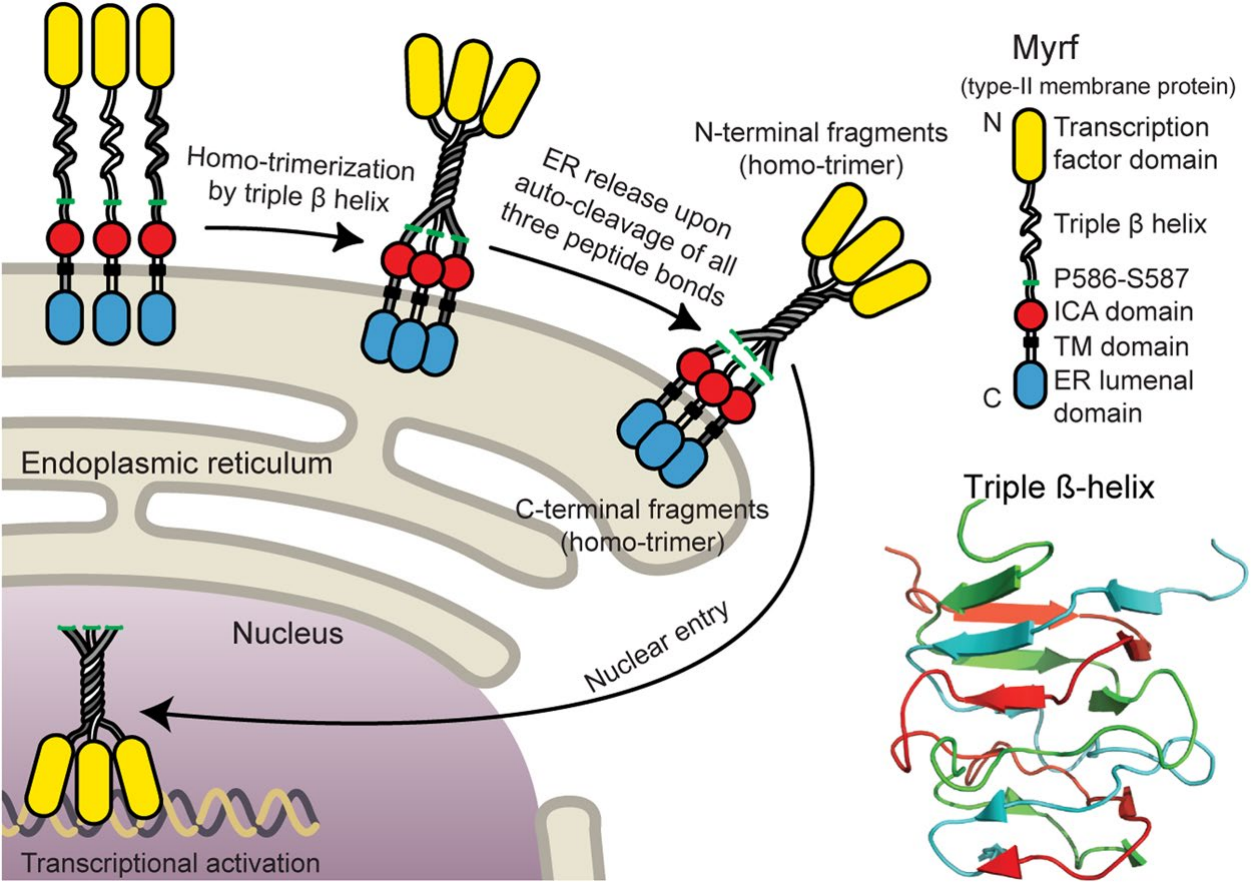
The schematic of Myrf auto-cleavage. The triple β-helix was taken from a crystal structure (PDB ID: 3GW6). Image rendered by PyMOL (The PyMOL Molecular Graphics System Version 1.7 Schrödinger LLC.).

Myrf is generated as a type-II membrane protein in the endoplasmic reticulum (ER)^4–6^. The Intramolecular Chaperone Auto-processing (ICA) domain is pivotal to the auto-cleavage of Myrf^4,5,20^. The ICA domain forms a homo-trimer, which as an intramolecular chaperone helps Myrf to form a homo-trimer in the ER membrane by chaperoning the formation of a triple β-helix (*the intramolecular chaperone function*, Fig. 1)^4,5,20–23^. As soon as the triple β-helix is completed, the ICA homo-trimer performs the auto-cleavage reactions that cleave the three P586-S587 peptide bonds connecting the homo-trimer of Myrf N-terminal fragments with the ICA homo-trimer^20^ (*the auto-processing function*, Fig. 1). Upon auto-cleavage, Myrf N-terminal homo-trimer is released from the ER membrane and enters the nucleus to function as a homo-trimer transcription factor^21,24,25^. The triple β-helix is an elaborate structure where three polypeptide chains come together and are tightly intertwined around a common three-fold axis (Fig. 1). Without the intramolecular chaperone function of the ICA homo-trimer, it cannot be formed on its own.

The core biochemical activity of the ICA homo-trimer – inducing homo-trimerization by triple β-helix and subsequently performing auto-cleavage – is conserved from human to virus^4,5,20–23^ and allows Myrf to work as a membrane-bound transcription factor. If deleterious mutations hit the ICA domain, its auto-cleavage function may be impaired, potentially abolishing the transcription factor function of Myrf. Several *MYRF* coding variants have been implicated in birth defects^13–19^. These anomalies commonly involve defects in heart, lung, and urogenital tract and are thought to constitute a novel syndrome. The observation that *Myrf* knockout led to embryonic lethality in mouse independent of OL development^1^ suggests that *MYRF* missense mutations observed in congenital anomalies may be pathogenic. However, none of the six *MYRF* missense mutations implicated in birth defects has been characterized, and it remains unknown whether and how they impair the transcription factor function of Myrf and, more importantly, how they are pathogenic for congenital anomalies in a heterozygous state. We have addressed this important issue, and this paper reports the results for two *MYRF* coding variants mapped to the ICA domain (V679A and R695H^13^). The other four *MYRF* missense mutations, which are all mapped to the DNA-binding domain, involve completely different molecular mechanisms than V679A and R695H, and their results will be reported later elsewhere.

## Results

### V679A and R695H mutations block the auto-cleavage of Myrf

**Figure 2:**
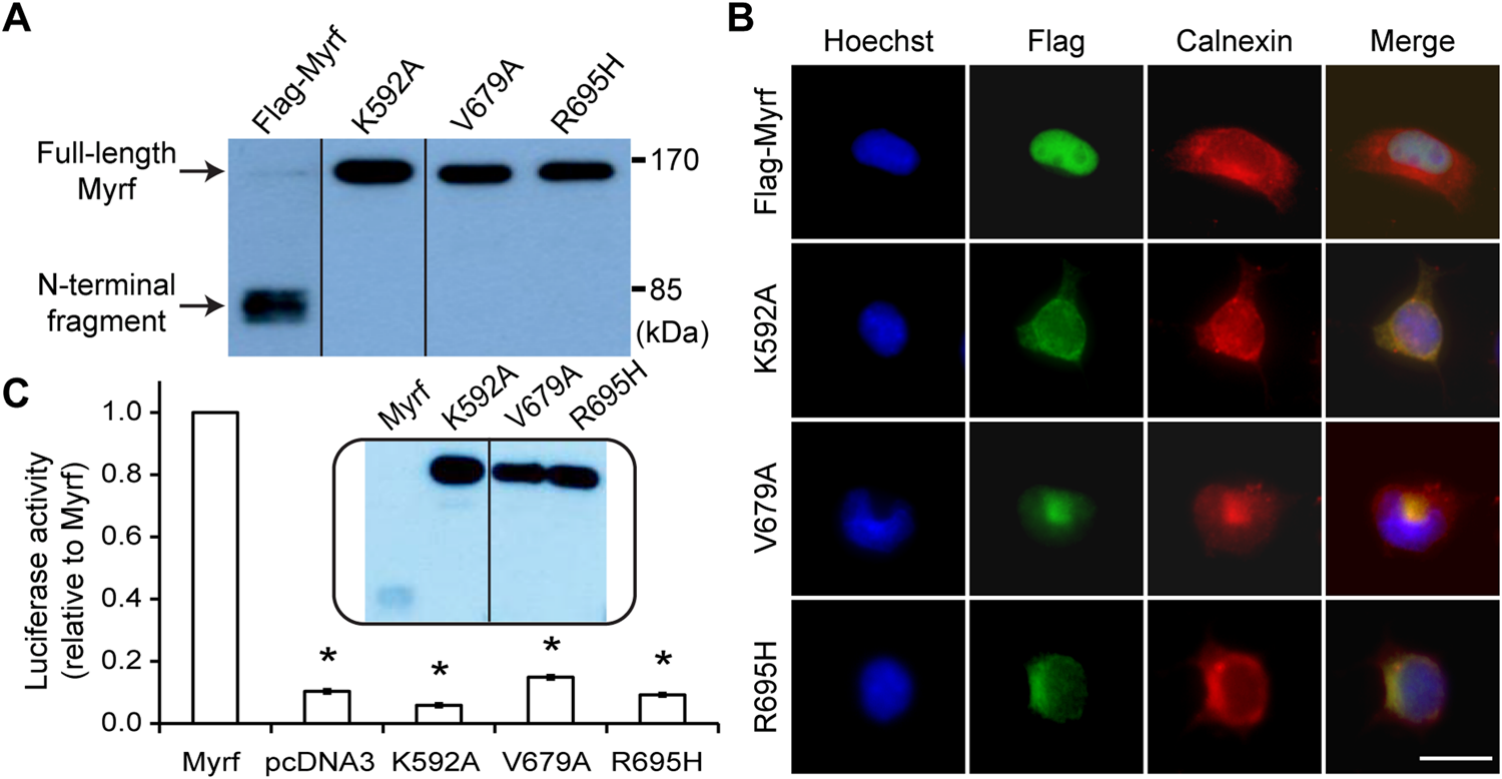
The auto-cleavage, subcellular localization, and transcriptional activity of Myrf-V679A and Myrf-R695H. (**A**) Western blot of Flag-Myrf-V679A and Flag-Myrf-R695H. Flag-Myrf-V679A and Flag-Myrf-R695H were expressed in Oli-neu cells, and whole cell lysates probed by Flag antibodies to determine the auto-cleavage of Flag-Myrf-V679A and Flag-Myrf-R695H. Flag-Myrf (wild-type Myrf) and Flag-Myrf-K592A (a known auto-cleavage mutant^4,5^) were analyzed in parallel as controls. These samples were run on the same gel, cut, and put together. The grouping of blots cropped from different parts of the same gel is indicated by dividing lines. The raw Western blot results are available in Fig. S1, where the cropped portions are marked by yellow boxes. (**B**) Immunofluorescence of Flag-Myrf-V679A and Flag-Myrf-R695H. Flag-Myrf-V679A and Flag-Myrf-R695H were expressed in Oli-neu cells, and the subcellular localization of their N termini determined by immunofluorescence with Flag antibodies. Flag-Myrf and Flag-Myrf-K592A were used for control experiments. Calnexin is an ER marker. Scale bar, 10 µm. (**C**) Luciferase assay of Flag-Myrf-V679A and Flag-Myrf-R695H with Rffl (a specific and sensitive Myrf luciferase reporter; see Methods for the detail). Flag-Myrf-V679A and Flag-Myrf-R695H were expressed in Oli-neu cells, and their transcriptional activity measured by the reporter activity of Rffl. Flag-Myrf, Flag-Myrf-K592A, and pcDNA3 (an empty vector) were used for control experiments. The reporter activity of Flag-Myrf was set to 1. Shown are the mean and standard error (n = 3). *p < 2.2 × 10^−4^ by two-tailed one sample Student’s *t* test with Bonferroni correction. Inset is the Western blot of one set of luciferase assay samples showing comparable protein expression. These samples were run on the same gel, cut, and put together. The grouping of blots cropped from different parts of the same gel is indicated by the dividing line. The raw Western blot results are available in Fig. S2, where the cropped portions are marked by yellow boxes.

V679A and R695H (according to NM_001127392.2^13^, also mapping to the same positions for the mouse Myrf used for the current study) were originally identified by an exome sequencing study of congenital diaphragmatic hernia^13^, a relatively common life-threatening birth defect (about 1 in 3000 live births affected). Congenital diaphragmatic hernia is caused by the defective formation of the diaphragm that results in the protrusion of abdomen viscera into the thoracic cavity. V679A and R695H are not found in nominally healthy individuals according to the NHLBI GO Exome Sequencing Project, the 1000 Genomes Project^26^, and the ExAC database^27^. Thus, they have been assumed to be deleterious. The fact that V679A and R695H are mapped to the ICA domain prompted us to hypothesize that they may cripple the ICA domain, abrogating the auto-cleavage and transcription factor function of Myrf. To test this hypothesis, we performed Western blot. Oli-neu cells, a widely used OL cell line^28^, were transfected with Flag-Myrf-V679A (Myrf-V679A with an N-terminal Flag tag) and Flag-Myrf-R695H (Myrf-R695H with an N-terminal Flag tag). Flag-Myrf (wild-type Myrf) and Flag-Myrf-K592A (a mutant that does not undergo auto-cleavage due to the mutation of the catalytic lysine residue^4,5,20^) were used for control experiments. Whole cell lysates were subject to immunoblotting with Flag antibodies. Flag-Myrf underwent auto-cleavage efficiently such that full-length Myrf is almost invisible (Fig. 2A)^29^. In contrast, Flag-Myrf-V679A and Flag-Myrf-R695H failed to do so (Fig. 2A). In fact, they were indistinguishable from Flag-Myrf-K592A in this regard. These Western blot results suggest that Flag-Myrf-V679A and Flag-Myrf-R695H would remain in the ER, like Flag-Myrf-K592A^4,5^. To check this, we transfected Oli-neu cells with the Myrf constructs and performed immunocytochemistry with Flag antibodies. The N terminus of Flag-Myrf was almost exclusively found in the nucleus (Fig. 2B), consistent with the nuclear translocation of Myrf N-terminal fragments upon auto-cleavage^4,5^. However, the N termini of Flag-Myrf-V679A and Flag-Myrf-R695H remained in the ER (Fig. 2B), in line with their auto-cleavage failure. Taken together, these data demonstrate that V679A and R695H block the auto-cleavage of Myrf.

### V679A and R695H mutations abrogate the transcriptional activity of Myrf

The above immunoblotting and immunofluorescence results predict that V679A and R695H would abrogate the transcriptional activity of Myrf. To test this hypothesis, we performed a luciferase assay in Oli-neu cells with Rffl. Rffl is a well-characterized Myrf luciferase reporter^5,21,29^, which was generated by cloning a Myrf ChIP-seq peak in the *Rffl* locus (rn4 chr10:71034166-71034749) into pGL3-promoter. Flag-Myrf significantly elevated the reporter activity of Rffl, as reported before^5,21,29^. In contrast, Flag-Myrf-V679A and Flag-Myrf-R695H failed to do so (Fig. 2C). The reporter activity induced by them was as low as that induced by Flag-Myrf-K592A and pcDNA3 (Fig. 2C). Western blot analysis of the luciferase assay samples showed that the mutant *Myrf* constructs were expressed well (Fig. 2C), ruling out the trivial possibility that no transcriptional activity for mutant Myrf species was due to poor protein expression. Collectively, these results indicate that V679A and R695H abolish the transcriptional activity of Myrf, which is consequent to the auto-cleavage failure.

### Molecular basis for the mutational effect of V679A

**Figure 3:**
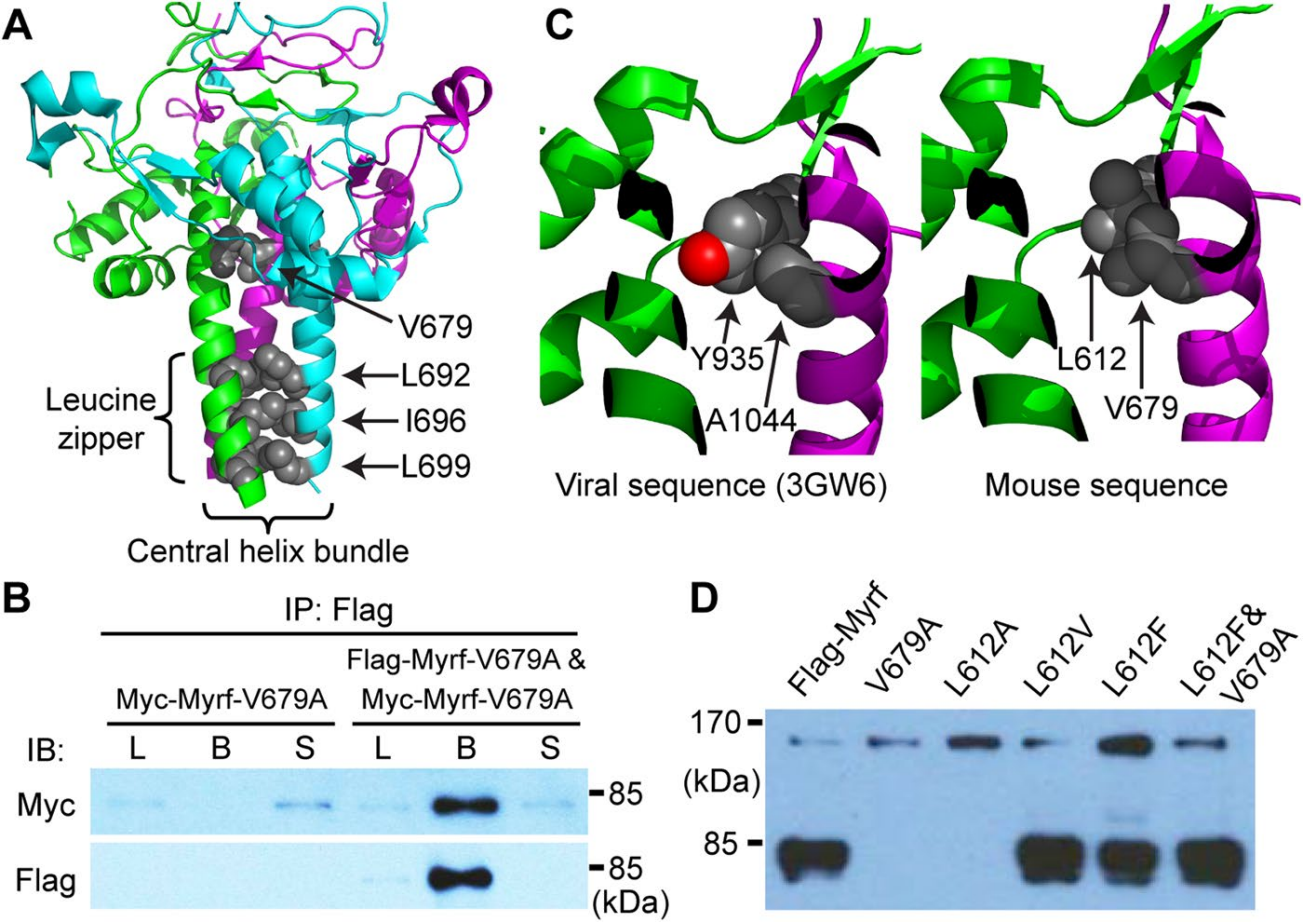
The molecular mechanism underlying the mutational effect of V679A. (**A**) The ICA homo-trimer crystal structure (PDB ID: 3GW6). Image rendered by PyMOL. (**B**) Homo-oligomerization of Myrf-V679A. Myc-Myrf-V679A was transfected into Oli-neu cells, either alone (for the left three lanes) or together with Flag-Myrf-V679A (for the right three lanes). Whole cell lysates were subject to immunoprecipitation with Flag beads. The three fractions of immunoprecipitation (L, B, S) were probed by Myc and Flag antibodies. L: whole cell lysate used for immunoprecipitation. B: bead fraction after immunoprecipitation. S: supernatant fraction after immunoprecipitation. IP: immunoprecipitation. IB: immunoblotting. The raw Western blot results are available in Fig. S3 (Myc) and Fig. S4 (Flag), where the cropped portions are marked by yellow boxes. (**C**) Molecular modeling of V679 and L612. Sequence alignment indicates that Myrf-V679 and Myrf-L612 correspond to A1044 and Y935 of the phage ICA domain (PDB ID: 3GW6). The crystal structure shows that A1044 packs against the aromatic ring of Y935. Molecular modeling by PyMOL suggests that V679 and L612 can form a similar inter-chain packing interaction. (**D**) Western blot of Myrf variants. Flag-Myrf and its variants (Flag-Myrf-V679A, Flag-Myrf-L612A, Flag-Myrf-L612V, Flag-Myrf-L612F, and Flag-Myrf-L612F&V679A) were expressed in Oli-neu cells, and whole cell lysates probed by Flag antibodies to determine the auto-cleavage of Myrf variants. The raw Western blot results are available in Fig. S5, where the cropped portions are marked by yellow boxes.

Our previous study has shown that the auto-cleavage function of the ICA homo-trimer is exquisitely sensitive to its correct conformation^4^. Even seemingly subtle mutations can cripple it, as illustrated by mutations affecting the leucine zipper of the ICA homo-trimer. The crystal structure of the ICA homo-trimer (PDB ID: 3GW6^20^) reveals a central helix bundle (Fig. 3A), which is sustained by the leucine zipper. The helix bundle mediates a myriad of inter-chain interactions and is critical to the correct assembly of the ICA homo-trimer. When any of the leucine zipper residues (L692, I696, and L699) is replaced by alanine (Fig. 3A), the ICA domain is unable to carry out the auto-cleavage reactions, even though it still forms a homo-oligomer^4^. Thus, a main mechanism of deleterious ICA domain mutations seems to prevent the ICA homo-trimer from adopting a correct conformation required for the auto-cleavage function. The crystal structure shows that V679 is situated at the head of the helix bundle (Fig. 3A), suggesting that V679A may work in a similar way to the leucine zipper mutants. To test this hypothesis, we performed immunoprecipitation experiments. Myc-Myrf-V679A was transfected into Oli-neu cells, either alone or together with Flag-Myrf-V679A. Cell lysates were subject to immunoprecipitation with Flag beads. When expressed alone, Myc-Myrf-V679A did not bind to Flag beads (Fig. 3B). When co-expressed with Flag-Myrf-V679A, however, it bound to Flag beads (Fig. 3B). These results indicate that the V679A mutation does not block the homo-oligomerization of the ICA domain, suggesting that it disrupts the auto-cleavage function of the ICA homo-trimer by interfering with its delicate homo-trimeric conformation. This conclusion is corroborated by the rescue of the auto-cleavage defect of Myrf-V679A by a compensatory mutation L612F (see below).

To gain insight into the molecular mechanism underlying the mutational effect of Myrf-V679A, we examined the ICA homo-trimer crystal structure^20^. V679 of the mouse Myrf corresponds to A1044 of the phage ICA domain. The crystal structure shows that A1044 of one chain tightly packs against the aromatic ring of Y935 of another chain (Fig. 3C left). Molecular modeling by PyMOL suggests that V679 and L612 of the mouse Myrf can make a similar inter-chain packing interaction (Fig. 3C right). This inter-chain packing interaction is expected to be crucial for the ICA homo-trimer. The V679A mutation may destabilize the ICA homo-trimer by disrupting this inter-chain packing interaction. To determine whether a defective packing interaction between V679 and L612 contributes to the mutational effect of V679A, we tested two predictions from the molecular modeling analysis. First, L612 is predicted to be as crucial as V679 for the inter-chain packing interaction. Thus, replacing L612 with less bulky amino acids may disturb the ICA homo-trimer, inhibiting its auto-cleavage function. In support of this hypothesis, we found that while replacing L612 with valine is tolerated, its replacement with alanine prevents the auto-cleavage of Myrf (Fig. 3D). Second, V679A may be rescued by replacing L612 with a bulkier hydrophobic residue. Consistently, while the L612F mutation was neutral by itself, it was able to restore the auto-cleavage of Myrf-V679A (Fig. 3D). These results lend support to our hypothesis that V679 and L612 form a crucial inter-chain packing interaction for the ICA homo-trimer to perform the auto-cleavage function.

### Molecular basis for the mutational effect of R695H

**Figure 4:**
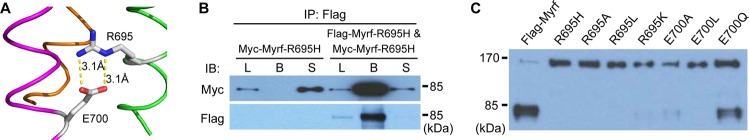
The molecular mechanism underlying the mutational effect of R695H. (**A**) Molecular modeling by PyMOL suggests that R695 and E700 are close to each other such that they can form two inter-chain hydrogen bonds. Å: 10^−1^ nm. (**B**) Homo-oligomerization of Myrf-R695H. Myc-Myrf-R695H was transfected into Oli-neu cells, either alone (for the left three lanes) or together with Flag-Myrf-R695H (for the right three lanes). Whole cell lysates were subject to immunoprecipitation with Flag beads. The three fractions of immunoprecipitation (L, B, S) were probed by Myc and Flag antibodies. L: whole cell lysate used for immunoprecipitation. B: bead fraction after immunoprecipitation. S: supernatant fraction after immunoprecipitation. IP: immunoprecipitation. IB: immunoblotting. The raw Western blot results are available in Fig. S6 (Myc) and Fig. S4 (Flag), where the cropped portions are marked by yellow boxes. (**C**) Western blot of Myrf variants. Flag-Myrf and its variants (Flag-Myrf-R695H, Flag-Myrf-R695A, Flag-Myrf-R695L, Flag-Myrf-R695K, Flag-Myrf-E700A, Flag-Myrf-E700L, and Flag-Myrf-E700Q) were expressed in Oli-neu cells, and whole cell lysates probed by Flag antibodies to determine the auto-cleavage of Myrf variants. The raw Western blot results are available in Fig. S7, where the cropped portions are marked by yellow boxes.

R695 is located in the central helix bundle (Fig. 4A), neighboring the leucine zipper (L692, I696, and L699). Molecular modeling by PyMOL suggests that R695 may form two inter-chain hydrogen bonds with E700 (Fig. 4A), and that the inter-chain hydrogen bonds may be critical to the ICA homo-trimer. Replacement of R695 by histidine is expected to disrupt the presumed inter-chain hydrogen bonds. Thus, akin to V679A, R695H is predicted to impair the auto-cleavage function of the ICA homo-trimer by interfering with its fine homo-trimeric conformation. To test this hypothesis, we performed immunoprecipitation experiments. Myc-Myrf-R695H was transfected into Oli-neu cells, either alone or together with Flag-Myrf-R695H. Cell lysates were subject to immunoprecipitation with Flag beads. When expressed alone, Myc-Myrf-R695H did not bind to Flag beads (Fig. 4B). When co-expressed with Flag-Myrf-R695H, however, it bound to Flag beads (Fig. 4B), lending support to our hypothesis that Myrf-R695H is able to form homo-oligomers.

To determine whether the presumed hydrogen bonds between R695 and E700 contribute to the mutational effect of R695H, we tested several predictions from the molecular modeling analysis. First, replacing R695 with alanine or leucine would be as detrimental to the auto-cleavage of Myrf as the R695H mutation. Second, the replacement of R695 with lysine may be partially tolerated because lysine can serve as a hydrogen bond donor. Third, E700 would be as important as R695 for the auto-cleavage of Myrf, and replacing E700 with alanine or leucine would prevent the auto-cleavage of Myrf. Fourth, exchanging E700 with glutamine may be partially tolerated because glutamine can serve as a hydrogen bond acceptor. We found that R695A, R695L, E700A, and E700L block the auto-cleavage of Myrf in support of our hypothesis (Fig. 4C). The E700Q mutation was partially tolerated. However, the R695K mutation was not tolerated. Overall, these results support our hypothesis that R695 and E700 form inter-chain hydrogen bonds, whose disruption underlies the mutational effect of R695H.

### V679A and R695H are not dominant negatives

**Figure 5:**
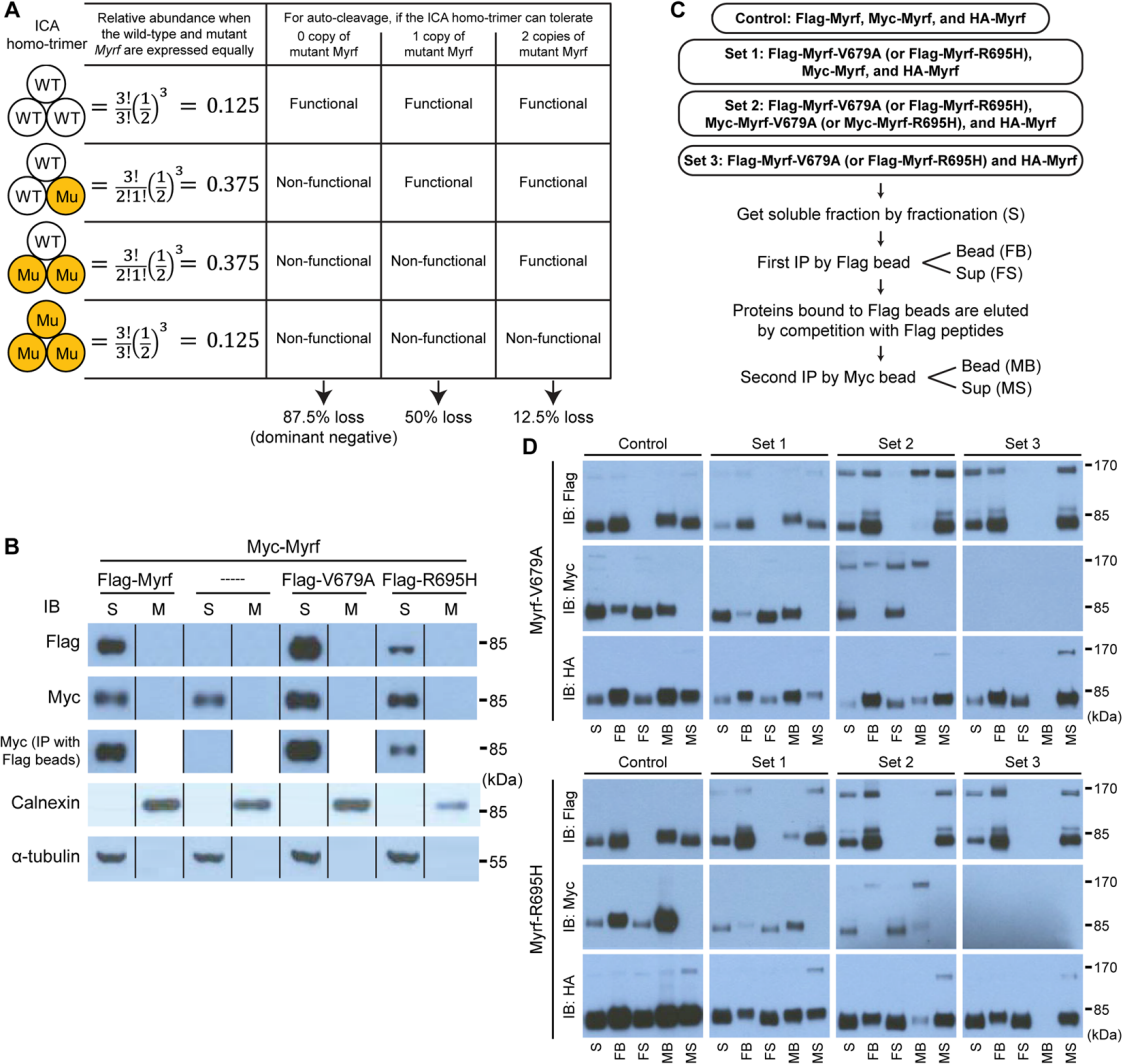
Fractionation and immunoprecipitation of wild-type and mutant Myrf N-terminal fragments. (**A**) The relative abundance of distinct ICA homo-trimer species when wild-type and mutant Myrf proteins are present in equal abundance. The fraction of disabled ICA homo-trimers depending on the number of the mutant Myrf tolerated by the ICA homo-trimer for its auto-cleavage function. (**B**) The auto-cleavage of mutant Myrf when complexed with two copies of wild-type Myrf. Myc-Myrf was transfected into HEK293FT cells, either alone (-----) or together with Flag-Myrf, Flag-Myrf-V679A, or Flag-Myrf-R695H. Whole cell lysates were separated into soluble (“S”) and membrane (“M”) fractions by fractionation. Immunoblotting with calnexin (an ER membrane protein) and α-tubulin indicated a good separation (the bottom two rows). The M and S fractions were blotted for Myrf N-terminal fragments with Flag and Myc antibodies (the top two rows). To determine physical association between wild-type and mutant Myrf N-terminal fragments, the S fractions were subject to immunoprecipitation with Flag beads, which were blotted for Myc-Myrf N-terminal fragments with Myc antibodies (the middle row). IP: immunoprecipitation. IB: immunoblotting. These samples were run on the same gel, cut, and put together. The grouping of blots cropped from different parts of the same gel is indicated by dividing lines. The raw Western blot results are available in Fig. S8 (Flag), Fig. S9 (Myc), Fig. S10 (calnexin), and Fig. S11 (α-tubulin), where the cropped portions are marked by yellow boxes. (**C**) Sequential immunoprecipitation to determine the maximum number of Myrf-V679A or Myrf-R695H that is tolerated by the ICA homo-trimer for its auto-cleavage function. For each mutant Myrf (Myrf-V679A and Myrf-R695H), 4 sets of plasmids were expressed in HEK293FT cells. After cells were broken by homogenization, the soluble fraction was obtained by fractionation (“S”). It was subject to immunoprecipitation with Flag beads, yielding the bead and sup fractions (“FB” and “FS”). Proteins bound to Flag beads were eluted by competition with Flag peptides. The eluate was subject to another round of immunoprecipitation with Myc beads, yielding the bead and sup fractions (“MB” and “MS”). (**D**) The five fractions (“S”, “FB”, “FS”, “MB”, and “MS”) from the sequential immunoprecipitation were blotted for full-length Myrf and Myrf N-terminal fragments with Flag, Myc, and HA antibodies. For each of the 6 immunoblots, the loading ratio among the five fractions was kept constant across the four sets for an objective comparison. IB: immunoblotting.

Since the ICA domain works as a homo-trimer for the auto-cleavage of Myrf, one copy of a *MYRF* allele encoding MYRF-V679A or MYRF-R695H does not necessarily mean 50% loss of function. If a mutant *MYRF* allele is expressed at a comparable level to the wild-type one, a likely scenario for a heterozygote^14^, 12.5% ( = 0.5^3^), 37.5% ( = 3 × 0.5^3^), 37.5% ( = 3 × 0.5^3^), and 12.5% ( = 0.5^3^) of ICA homo-trimers would contain 0, 1, 2, and 3 copies of the mutant ICA, respectively (Fig. 5A). V679A and R695H were assumed to be present as a heterozygote in patients because they were *de novo* mutations^13^. For Myrf N-terminal fragments to be released from the ER membrane as a homo-trimer, all three Myrf polypeptide chains must undergo auto-cleavage at the P586-S587 peptide bond (Fig. 1). If any fails to undergo auto-cleavage, no fragment is released from the ER membrane, including the cleaved one, because homo-trimerization by the triple β-helix is almost irreversible (Fig. 1). If the ICA homo-trimer cannot tolerate even 1 copy of the mutant ICA for the three auto-cleavage reactions, 87.5% of ICA homo-trimers would be disabled (Fig. 5A), making the mutant Myrf work as a dominant negative. Our previous study showed that this is the case for Myrf-K592A^21^. If the ICA homo-trimer can tolerate one, but not two, copy of the mutant ICA, 50% of ICA homo-trimers would not be able to release Myrf N-terminal homo-trimers from the ER membrane (Fig. 5A). If the ICA homo-trimer can tolerate as many as two copies of the mutant ICA, only 12.5% of ICA homo-trimers would be defective.

Since V679A and R695H have been implicated in congenital anomalies, we wondered whether they work as a dominant negative, the worst possible scenario. To test it, we performed fractionation and immunoprecipitation experiments. Myc-Myrf was transfected into HEK293FT cells, together with Flag-Myrf-V679A or Flag-Myrf-R695H at the ratio of 19:1 to maximize the abundance of ICA homo-trimers that contain 1 mutant and 2 wild-type copies. At this ratio, more than 95% of the mutant Myrf are expected to be associated with 2 wild-type copies. For control experiments, Myc-Myrf was transfected into HEK293FT cells, either alone or together with Flag-Myrf (at the same ratio of 19:1). After cells were broken by homogenization, whole cell lysates were separated into soluble and membrane fractions by fractionation^4^. Immunoblotting with calnexin (an ER membrane protein) and α-tubulin indicated a good separation (“S” and “M” for the soluble and membrane fractions, respectively, Fig. 5B). Remarkably, Flag-Myrf-V679A and Flag-Myrf-R695H underwent auto-cleavage in our experimental condition, which maximizes its association with 2 wild-type copies (Fig. 5B). Further, their N-terminal fragments were found in the soluble fraction, but not in the membrane fraction, like wild-type Myrf N-terminal fragments (Fig. 5B). These observations indicate that the ICA homo-trimer that contains 1 copy of Flag-Myrf-V679A or Flag-Myrf-R695H and 2 wild-type copies is capable of the three auto-cleavage reactions that are required for the ER release of Myrf N-terminal fragments as a homo-trimer. To confirm that the N-terminal fragment of Flag-Myrf-V679A or Flag-Myrf-R695H released from the ER membrane is physically associated with wild-type Myrf N-terminal fragments, the soluble fractions were subject to immunoprecipitation with Flag beads. It showed that the N-terminal fragments of Flag-Myrf-V679A or Flag-Myrf-R695H in the soluble fraction are indeed complexed with those of wild-type Myrf (Fig. 5B). The specificity of the immunoprecipitation procedure was validated by the soluble fraction where there was no Flag-Myrf expressed (---- in Fig. 5B). Collectively, these results indicate that Myrf-V679A and Myrf-R695H do not work as a dominant negative. In other words, the ICA homo-trimer can tolerate at least one copy of Myrf-V679A or Myrf-R695H for the auto-cleavage function.

### The ICA homo-trimer can tolerate at most one copy of Myrf-V679A or Myrf-R695H

The preceding fractionation results prompted us to ask whether the ICA homo-trimer can tolerate two copies of Myrf-V679A or Myrf-R695H. To test it, we performed sequential immunoprecipitation experiments for Myrf-V679A and Myrf-R695H. For each mutant, we expressed 4 sets of plasmids in HEK293FT cells (Fig. 5C). The control set expressed three wild-type Myrf species (Flag-Myrf, Myc-Myrf, and HA-Myrf). Set 1 expressed one mutant and two wild-type Myrf species (Flag-Myrf-V679A (or Flag-Myrf-R695H), Myc-Myrf, and HA-Myrf). Set 2 consisted of two mutant and one wild-type Myrf species (Flag-Myrf-V679A (or Flag-Myrf-R695H), Myc-Myrf-V679A (or Myc-Myrf-R695H), and HA-Myrf). Set 3, which comprised Flag-Myrf-V679A (or Flag-Myrf-R695H) and HA-Myrf, was to check the specificity of immunoprecipitation with Myc beads. After cells were broken by homogenization, the soluble fraction was obtained by fractionation (“S” in Fig. 5C). It was subject to immunoprecipitation with Flag beads, yielding the bead and sup fractions (“FB” and “FS” in Fig. 5C). Proteins bound to Flag beads were eluted by competition with Flag peptides. The eluate was subject to another round of immunoprecipitation with Myc beads, yielding the bead and sup fractions (“MB” and “MS” in Fig. 5C). Our previous study used a similar sequential immunoprecipitation technique to demonstrate that Myrf N-terminal fragments exist as homo-trimers^21^.

The five fractions (“S”, “FB”, “FS”, “MB”, and “MS”) were blotted for full-length Myrf and Myrf N-terminal fragments with Flag, Myc, and HA antibodies (Fig. 5D). For each of the 6 immunoblots, the loading ratio among the five fractions was kept constant across the four sets for an objective comparison. For the control set, we were able to detect the N-terminal fragments of Flag-Myrf, Myc-Myrf, and HA-Myrf in the MB fraction (Fig. 5D), consistent with its homo-trimerization^21,24,25^. The specificity of the second Myc immunoprecipitation step was confirmed by the lack of immunoblot signals in the MB fraction for the Set 3 samples. We could also detect the N-terminal fragments of Flag-Myrf-V679A (or Flag-Myrf-R695H), Myc-Myrf, and HA-Myrf in the MB fraction for the Set 1 samples (Fig. 5D), indicating that the ICA homo-trimer can tolerate one copy of Myrf-V679A or Myrf-R695H for the auto-cleavage function. This is in line with the fractionation result in Fig. 5B. However, we note that the auto-cleavage efficiency of the ICA homo-trimer that contains one copy of Myrf-R695H was not as high as that of the wild-type ICA homo-trimer whereas the ICA homo-trimer that contains one copy of Myrf-V679A underwent auto-cleavage as efficiently as the wild-type ICA homo-trimer. The same trend is also observed in the fractionation results (Fig. 5B). These observations suggest that Myrf-R695H is not tolerated as well as Myrf-V679A by the ICA homo-trimer. For the Set 2 samples, we could not detect the N-terminal fragments of Flag-Myrf-V679A (or Flag-Myrf-R695H) and Myc-Myrf-V679A (or Myc-Myrf-R695H) in the MB fraction (Fig. 5D). These results demonstrate that the ICA homo-trimer cannot tolerate two mutant copies for the three auto-cleavage reactions. Taken together, we conclude that the ICA homo-trimer can tolerate at most one copy of Myrf-V679A or Myrf-R695H for the auto-cleavage function.

## Discussion

Myrf is a pleiotropic membrane-bound transcription factor, playing a critical role in diverse organisms ranging from human to slime mold^1–7^. The ICA domain is essential for the auto-cleavage of Myrf, which liberates its N-terminal fragments from the ER membrane as a homo-trimer^4,5^. The homo-trimer of Myrf N-terminal fragments enters the nucleus to work as a homo-trimeric transcription factor^21,29^. Our current study shows that V679A and R695H, two *MYRF* coding variants implicated in congenital diaphragmatic hernia^13^, cripple the ICA domain, abrogating the auto-cleavage and transcription factor function of Myrf. The crystal structure of the ICA homo-trimer suggests potential molecular mechanisms for the mutational effects of V679A and R695H^20^. V679 appears to form an inter-chain packing interaction with L612 while R695 and E700 seem to make inter-chain hydrogen bonds. These inter-chain interactions are expected to be critical to the correct conformation of the ICA homo-trimer that is required for its auto-cleavage function. The V679A and R695H mutations would disrupt the inter-chain interactions, preventing the ICA homo-trimer from adopting the correct conformation required for the three auto-cleavage reactions. The V679-L612 packing interaction seems malleable because (1) the auto-cleavage defect of Myrf-V679A can be rescued by the L612F mutation and (2) they are mediated by A1044 and Y935 in the phage ICA domain, compensatory mutations that would preserve the packing interaction. The R695-E700 hydrogen bonds seem more rigid because conservative substitutions such as R695K and E700Q are not well tolerated. Further, our fractionation and sequential immunoprecipitation experiments reproducibly recovered less Myrf N-terminal homo-trimers for the ICA homo-trimer containing one copy of Myrf-R695H than for the one containing one copy of Myrf-V679A. Together with our previous study on the leucine zipper mutants of the ICA domain^4^, a common theme threading through deleterious ICA domain mutations is that they interfere with the fine conformation of the ICA homo-trimer to impair its auto-cleavage function.

According to the ExAC database, 11:61539012 TC, a variant *MYRF* allele that encodes a frameshift mutation (p.Ser264GlnfsTer74), is found in nominally healthy individuals at a frequency of 0.0006492 (about 6.5 in 10000), which is higher than that of congenital diaphragmatic hernia (about 1 in 3000). Given the location of the frameshift mutation, 11:61539012 TC must be a null allele. This allele was found as a heterozygote for all cases. Qi *et al*. dismissed this variant as “homo-polymer artifacts” without presenting any evidence^13^. As pointed out by Qi *et al*., 11:61539012 TC is found in a stretch of C’s, where sequencing errors often occur. It is possible that the high frequency of 11:61539012 TC is fully explained by sequencing errors. However, 11:61539012 TC was also detected by Garnai *et al*.^14^, suggesting that 11:61539012 TC may be real. The verity of 11:61539012 TC and its true frequency in nominally healthy individuals are important topics to be elucidated in the future. If we assume that 11:61539012 TC is a homo-polymer artifact, as suggested by Qi *et al*.^13^, *MYRF* may be haploinsufficient for embryonic development, implicating V679A and R695H in the pathogenesis of congenital anomalies. There are two ways for V679A and R695H to be pathogenic in a heterozygous state – haploinsufficiency and dominant negative. Through fractionation and sequential immunoprecipitation experiments, we found that the ICA homo-trimer can tolerate one copy of Myrf-V679A or Myrf-R695H for its auto-cleavage function, excluding the possibility that Myrf-V679A and Myrf-R695H are dominant negatives. Thus, if V679A and R695H were pathogenic in a heterozygous state for birth defects, it would be via haploinsufficiency of *MYRF*.

After the auto-cleavage of Myrf, Myrf C-terminal fragments remain in the ER membrane as a homo-trimer^4,21^ (Fig. 1). Little is known about the molecular function of Myrf C-terminal fragment and its role in the biological processes in which Myrf has been implicated. Being part of the ICA domain, V679 and R695 belong to Myrf C-terminal fragment. It is possible that V679A and R695H impair the unknown molecular function of Myrf C-terminal fragment in a dominant negative manner. For this to be true, however, one copy of the mutant ICA should be sufficient to disable the homo-trimeric complex of Myrf C-terminal fragments. This seems unlikely, though, because our work shows that the ICA homo-trimer containing one mutant copy is capable of a highly sophisticated molecular feat – performing the three auto-cleavage reactions for the ER release of Myrf N-terminal fragments as a homo-trimer. This is why we favor the conclusion that V679A and R695H are not dominant negatives.

## Methods

### Constructs

A *Myrf* cDNA that encodes the 1139-amino-acid-long mouse isoform^1^ was kindly provided by Dr. Ben Emery. V679A and R695H (according to NM_001127392.2^13^) map to the same positions for this mouse Myrf, which was used for all the experiments reported in this study. The cDNA was cloned into pcDNA3 with an N-terminal Flag, Myc, or HA tag by using the In-Fusion cloning kit from Clontech. Point mutations were introduced by a PCR-based method. Rffl was generated by cloning a rat genomic fragment (rn4 chr10:71034166-71034749) into pGL3-promoter (Promega)^5,21^. The sequence information of all constructs was verified by Sanger sequencing.

### Cell culture

Oli-neu cells were kept in a proliferating condition by supplementing the Sato media^30^ with PDGF (10 μg/mL), NT3 (1 μg/mL), CNTF (10 μg/mL), and NeuroCult™ SM1 Neuronal Supplement. They were maintained in a humidified 8% CO_2_ incubator at 37 °C. HEK293FT cells were cultured in Dulbecco’s modified Eagle’s medium supplemented with 10% fetal bovine serum and maintained in a humidified 5% CO_2_ incubator at 37 °C. Transient transfection was performed by using Lipofectamine 2000 as per the manufacturer’s instructions.

### Immunoblotting

Cells were rinsed once with PBS and lysed with 2X Laemmli Sample Buffer (Bio-Rad). Cell lysates were boiled at 95 °C for 5 min. Upon SDS-PAGE, proteins were transferred to PVDF and probed with horseradish peroxidase (HRP)-conjugated primary antibodies. The following dilutions were used for immunoblotting: mouse anti-FLAG HRP-conjugated (Sigma #A8592, 1:5000), mouse anti-HA HRP conjugated (Cell signaling #2999, 1:5000), mouse anti-c-Myc HRP-conjugated (Santa Cruz #sc-40, 1:2000), goat anti-calnexin (Santa Cruz #sc-6465, 1:500), and mouse anti-α-tubulin (Sigma #T9026, 1:50000).

### Immunofluorescence

Cells were fixed with 4% formaldehyde and permeabilized with 0.1% Triton X-100. Upon blocking with 1% BSA, they were incubated with primary antibodies diluted in the blocking buffer at 4 °C overnight, followed by incubation with fluorochrome-conjugated secondary antibodies. Nuclei were stained with Hoechst 33342 (Invitrogen). Fluorescence was visualized with a Leica DMi8 microscope with an ORCA-Flash4.0 sCMOS camera. Reagents used for immunofluorescence are as follows: monoclonal ANTI-FLAG M2 antibody (Sigma, 1:1000), goat anti-mouse calnexin antibody (Santa Cruz, 1:500), donkey anti-mouse antibody, Alexa Fluor 488 conjugate (ThermoFisher, 1:5000), and donkey anti-goat antibody, Alexa Fluor 594 conjugate (ThermoFisher, 1:5000).

### Immuoprecipitation

Cells grown on 150 mm culture dishes were rinsed once with PBS, and 500 μL of 2X Cell Lysis Buffer (Cell Signaling) was added to them. Cell lysates were sonicated and spun down at 14,000 *g* for 10 min at 4 °C. Cleared cell lysates were mixed with antibody-coated beads (Sigma) and incubated for 2 h at 4 °C on a rotating plate. The mix was spun down at 7,500 *g* for 30 s to separate it into supernatant and bead fractions. Sequential immunoprecipitation was performed as described previously^21^.

### Luciferase assay

Luciferase assay was performed by using the Promega dual luciferase reporter assay kit as per the manufacturer’s instructions. Cells were co-transfected with a Myrf construct, Rffl, and pRL-TK (an internal control). The ratio between firefly and renilla luciferase activities was taken as the transcriptional activity of the Myrf construct.

### Fractionation

Cells were swollen by incubation in a hypotonic buffer (10 mM HEPES, pH 7.9, 1.5 mM MgCl_2_ and 10 mM KCl) on ice. Swollen cells were disrupted using a Dounce-type homogenizer and centrifuged at 200 *g* for 5 min to obtain a supernatant fraction. The supernatant fraction was centrifuged at 17,000 *g* for 15 min at 4 °C to separate the soluble fraction from the membrane fraction.

## Supplementary information

Supplementary Information.

## Data Availability

The datasets generated and/or analyzed during the current study are available from the corresponding author on reasonable request.

## References

[1] Emery B (2009). Myelin gene regulatory factor is a critical transcriptional regulator required for CNS myelination. Cell.

[2] Russel S, Frand AR, Ruvkun G (2011). Regulation of the C. elegans molt by pqn-47. Dev. Biol..

[3] Senoo H, Wang HY, Araki T, Williams JG, Fukuzawa M (2012). An orthologue of the Myelin-gene Regulatory Transcription Factor regulates Dictyostelium prestalk differentiation. Int. J. Dev. Biol..

[4] Li ZH, Park Y, Marcotte EM (2013). A bacteriophage tailspike domain promotes self-cleavage of a human membrane-bound transcription factor, the myelin regulatory factor MYRF. PLoS Biol..

[5] Bujalka H (2013). MYRF is a membrane-associated transcription factor that autoproteolytically cleaves to directly activate myelin genes. PLoS Biol..

[6] Senoo H, Araki T, Fukuzawa M, Williams JG (2013). A new kind of membrane-tethered eukaryotic transcription factor that shares an auto-proteolytic processing mechanism with bacteriophage tail-spike proteins. J. Cell Sci..

[7] Meng J (2017). Myrf ER-bound transcription factors drive C. elegans synaptic plasticity via cleavage-dependent nuclear translocation. Developmental Cell.

[8] Zhang Y (2014). An RNA-sequencing transcriptome and splicing database of glia, neurons, and vascular cells of the cerebral cortex. J. Neurosci..

[9] Koenning M (2012). Myelin gene regulatory factor is required for maintenance of myelin and mature oligodendrocyte identity in the adult CNS. J. Neurosci..

[10] McKenzie IA (2014). Motor skill learning requires active central myelination. Science.

[11] Duncan GJ (2017). Myelin regulatory factor drives remyelination in multiple sclerosis. Acta Neuropathologica.

[12] Lonsdale J (2013). The Genotype-Tissue Expression (GTEx) project. Nat. Genet..

[13] Qi H (2018). De novo variants in congenital diaphragmatic hernia identify MYRF as a new syndrome and reveal genetic overlaps with other developmental disorders. PLoS Genet..

[14] Garnai SJ (2019). Variants in myelin regulatory factor (MYRF) cause autosomal dominant and syndromic nanophthalmos in humans and retinal degeneration in mice. PLoS Genet..

[15] Hamanaka, K. *et al*. MYRF haploinsufficiency causes 46,XY and 46,XX disorders of sex development: bioinformatics consideration. *Human Molecular Genetics* (2019). 10.1093/hmg/ddz066 30985895

[16] Pinz H (2018). De novo variants in Myelin regulatory factor (MYRF) as candidates of a new syndrome of cardiac and urogenital anomalies. Am. J. Med. Genet. Part. A.

[17] Jin SC (2017). Contribution of rare inherited and de novo variants in 2,871 congenital heart disease probands. Nat. Genet..

[18] Kurahashi H (2018). MYRF is associated with encephalopathy with reversible myelin vacuolization. Ann. Neurol..

[19] Rossetti, L. Z. *et al*. Review of the phenotypic spectrum associated with haploinsufficiency of MYRF. *American Journal of Medical Genetics Part A* (2019). 10.1002/ajmg.a.61182 PMC6557668 31069960

[20] Schulz EC (2010). Crystal structure of an intramolecular chaperone mediating triple-β-helix folding. Nat. Struct. Mol. Biol..

[21] Kim D (2017). Homo-trimerization is essential for the transcription factor function of Myrf for oligodendrocyte differentiation. Nucleic Acids Res..

[22] Muhlenhoff M, Stummeyer K, Grove M, Sauerborn M, Gerardy-Schahn R (2003). Proteolytic processing and oligomerization of bacteriophage-derived endosialidases. J. Biol. Chem..

[23] Schwarzer D, Stummeyer K, Gerardy-Schahn R, Muhlenhoff M (2007). Characterization of a novel intramolecular chaperone domain conserved in endosialidases and other bacteriophage tail spike and fiber proteins. J. Biol. Chem..

[24] Zhen X (2017). Crystal structure of the DNA-binding domain of Myelin-gene Regulatory Factor. Sci. Rep..

[25] Chen B, Zhu Y, Ye S, Zhang R (2018). Structure of the DNA-binding domain of human myelin-gene regulatory factor reveals its potential protein-DNA recognition mode. J. Struct. Biol..

[26] The 1000 Genomes Project Consortium. (2015). A global reference for human genetic variation. Nature.

[27] Lek M (2016). Analysis of protein-coding genetic variation in 60,706 humans. Nature.

[28] Jung M (1995). Lines of murine oligodendroglial precursor cells immortalized by an activated neu tyrosine kinase show distinct degrees of interaction with axons *in vitro* and *in vivo*. Eur. J. Neurosci..

[29] Choi J-o (2018). Elucidating the transactivation domain of the pleiotropic transcription factor Myrf. Sci. Rep..

[30] Emery B, Dugas JC (2013). Purification of oligodendrocyte lineage cells from mouse cortices by immunopanning. Cold Spring Harb. Protoc..

