# Supplementary material for: Functional mechanism and pathogenic potential of MYRF ICA domain mutations implicated in birth defects
Source: Sci Rep. 2020 Jan 21;10:814. doi: 10.1038/s41598-020-57593-8 (PMC6972908; doi:10.1038/s41598-020-57593-8)
Supplement: Supplementary file 1 — Supplementary Information. [file 41598_2020_57593_MOESM1_ESM.docx]

**Supplementary Information**

**Functional mechanism and pathogenic potential of MYRF ICA domain mutations**

**implicated in birth defects**

Hongjoo An^1^, Chuandong Fan^1^, Mohamed Sharif^1^, Dongkyeong Kim^1^,

Yannick Poitelon^2^, and Yungki Park^1,^*

^1^Hunter James Kelly Research Institute, Department of Biochemistry, Jacobs School of Medicine and Biomedical Sciences, State University of New York at Buffalo, Buffalo, NY 14203, USA

^2^Department of Neuroscience and Experimental Therapeutics, Albany Medical College, Albany, NY 12208, USA

*To whom correspondence should be addressed.

Tel: 1-716-881-7579; Fax: 1-716-849-6651; Email: [yungkipa@buffalo.edu](mailto:yungkipa@buffalo.edu)

**Figure S1**

The raw Western blot results for Figure 2A in the main text. Portions marked by yellow boxes are the ones shown in Figure 2A.

**
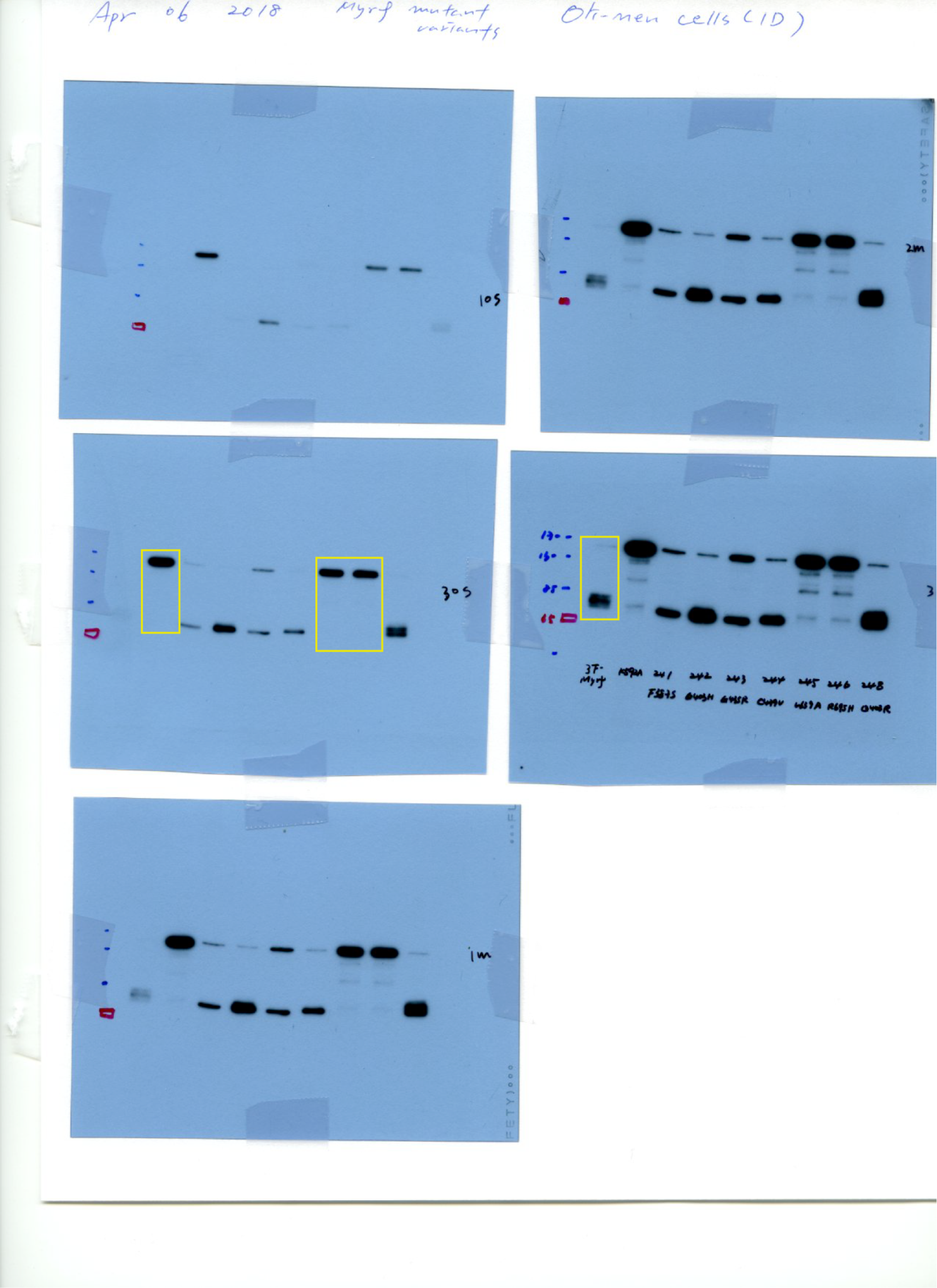
**

**Figure S2**

The raw Western blot results for Figure 2C in the main text. Portions marked by yellow boxes are the ones shown in Figure 2C.

**
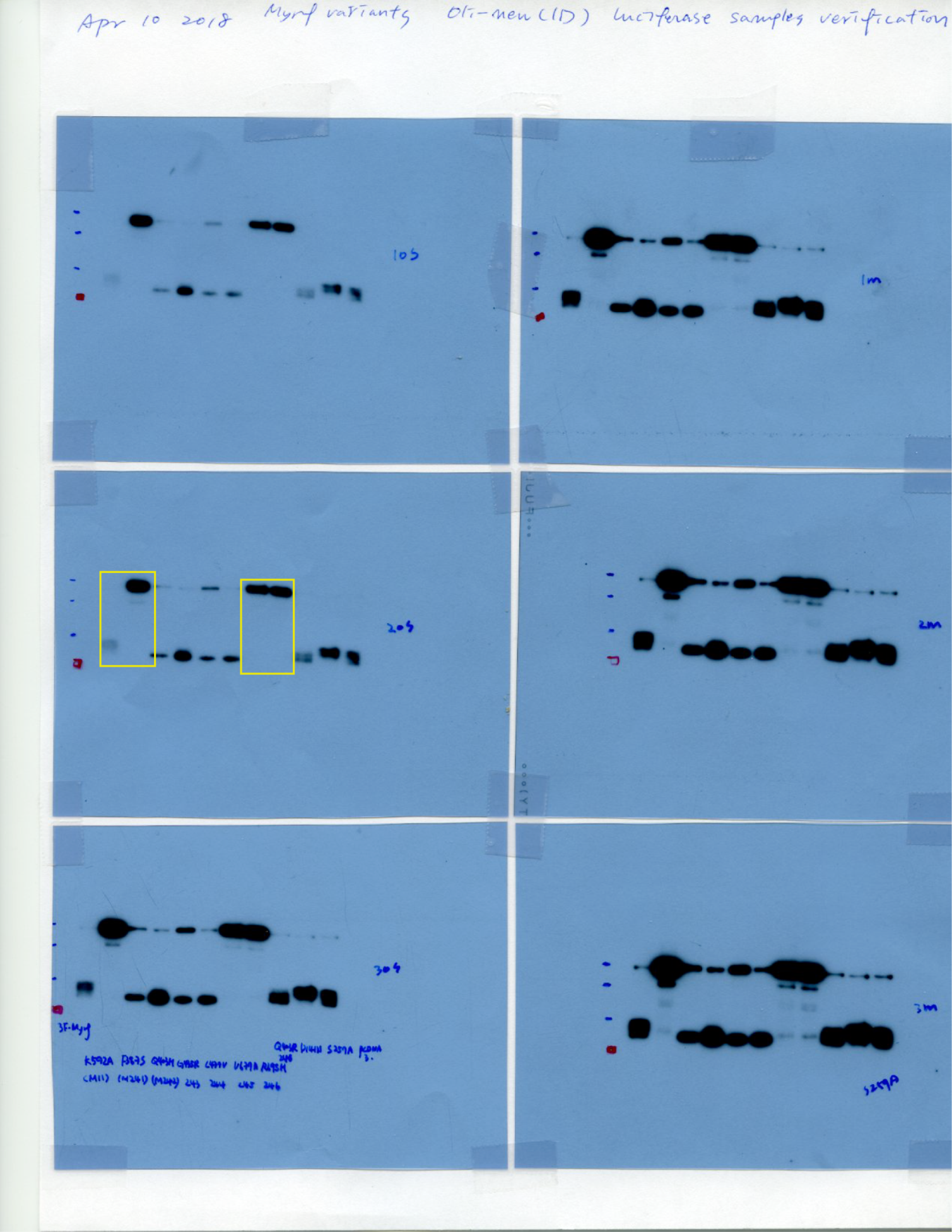
**

**Figure S3**

The raw Western blot results for Figure 3B in the main text. Portions marked by yellow boxes are the ones shown in Figure 3B.

**
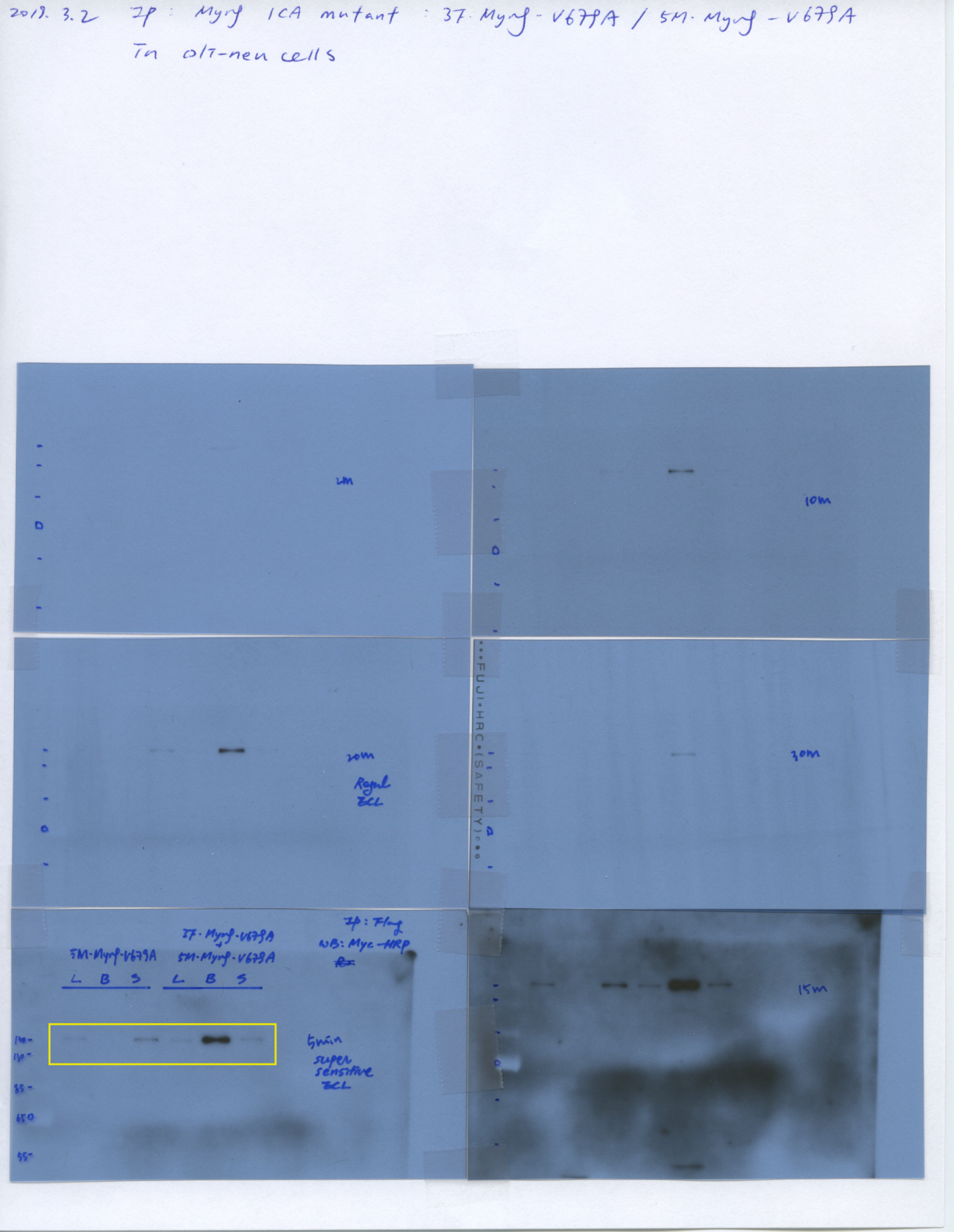
**

**Figure S4**

The raw Western blot results for Figures 3B and 4B in the main text. Portions marked by yellow boxes are the ones shown in Figures 3B and 4B.

**
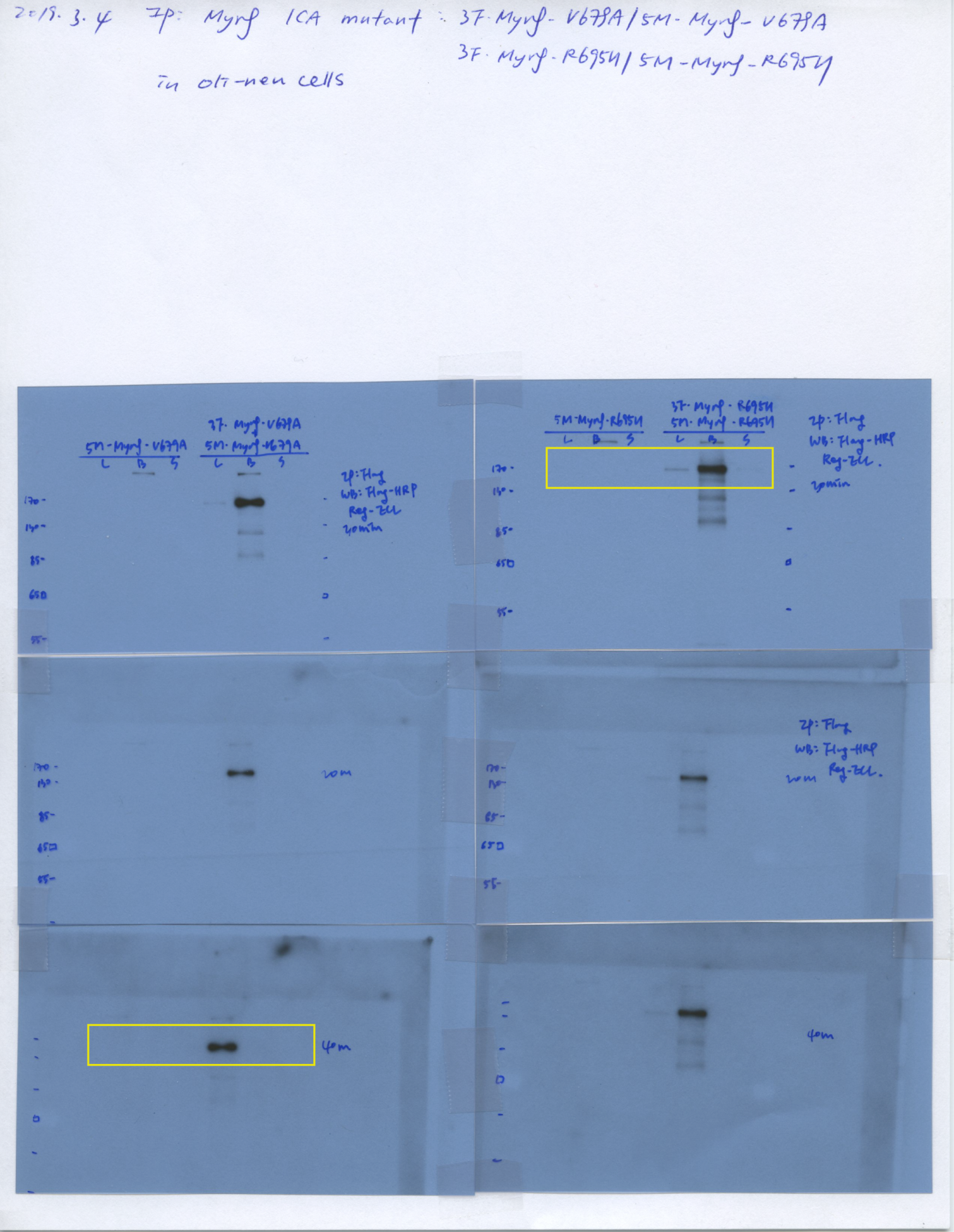
**

**Figure S5**

The raw Western blot results for Figure 3D in the main text. Portions marked by yellow boxes are the ones shown in Figure 3D.

**
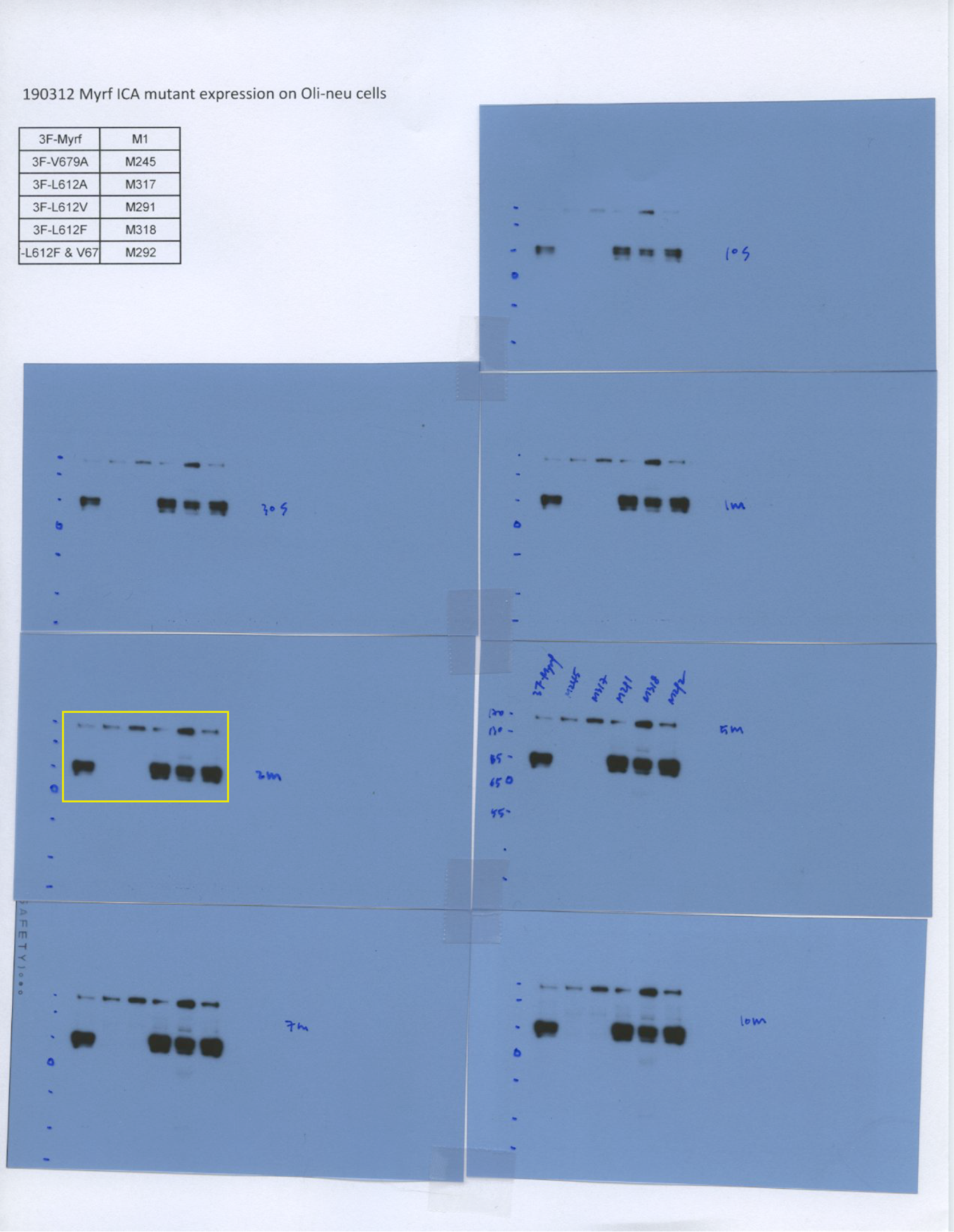
**

**Figure S6**

The raw Western blot results for Figure 4B in the main text. Portions marked by yellow boxes are the ones shown in Figure 4B.

**
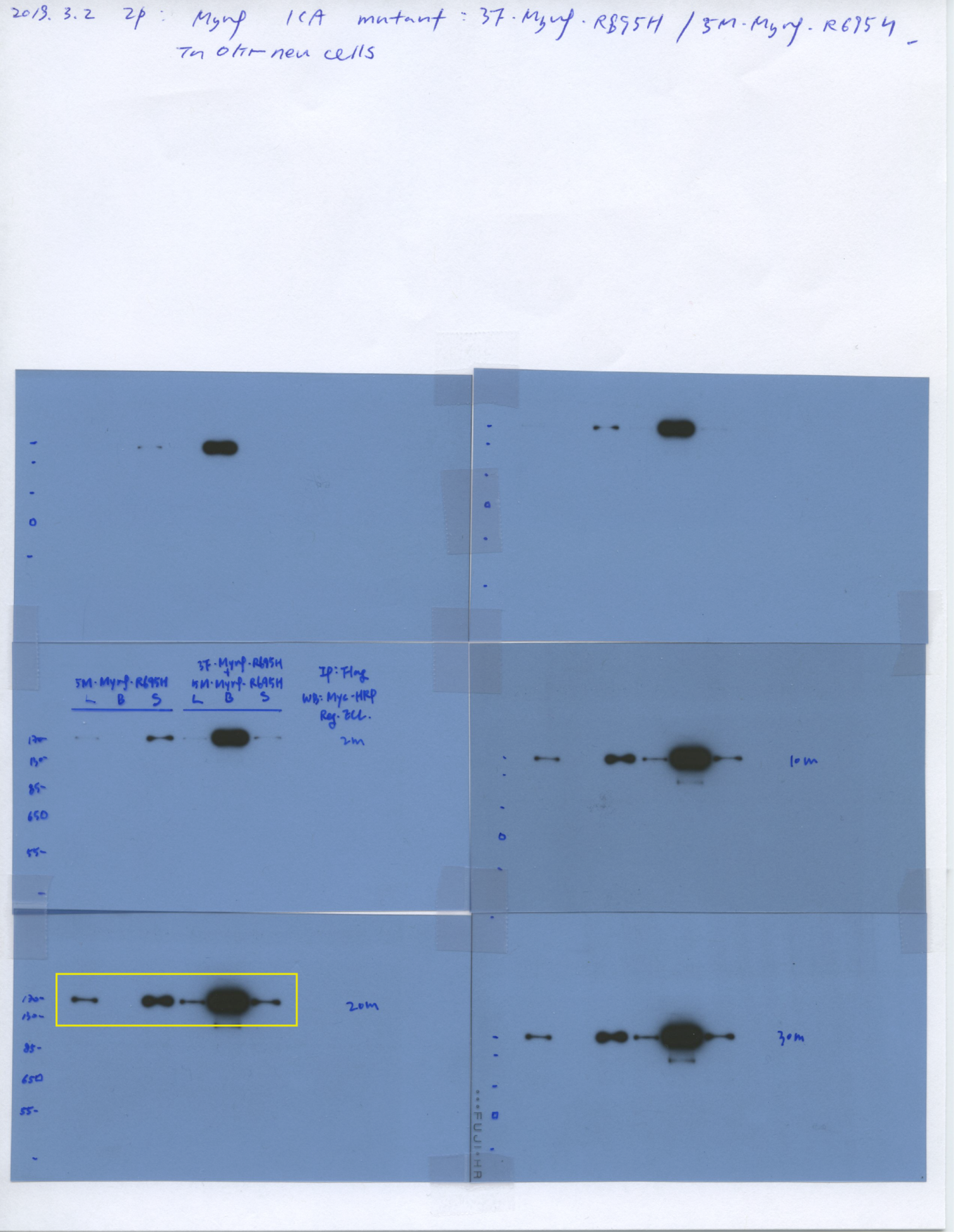
**

**Figure S7**

The raw Western blot results for Figure 4C in the main text. Portions marked by yellow boxes are the ones shown in Figure 4C.

**
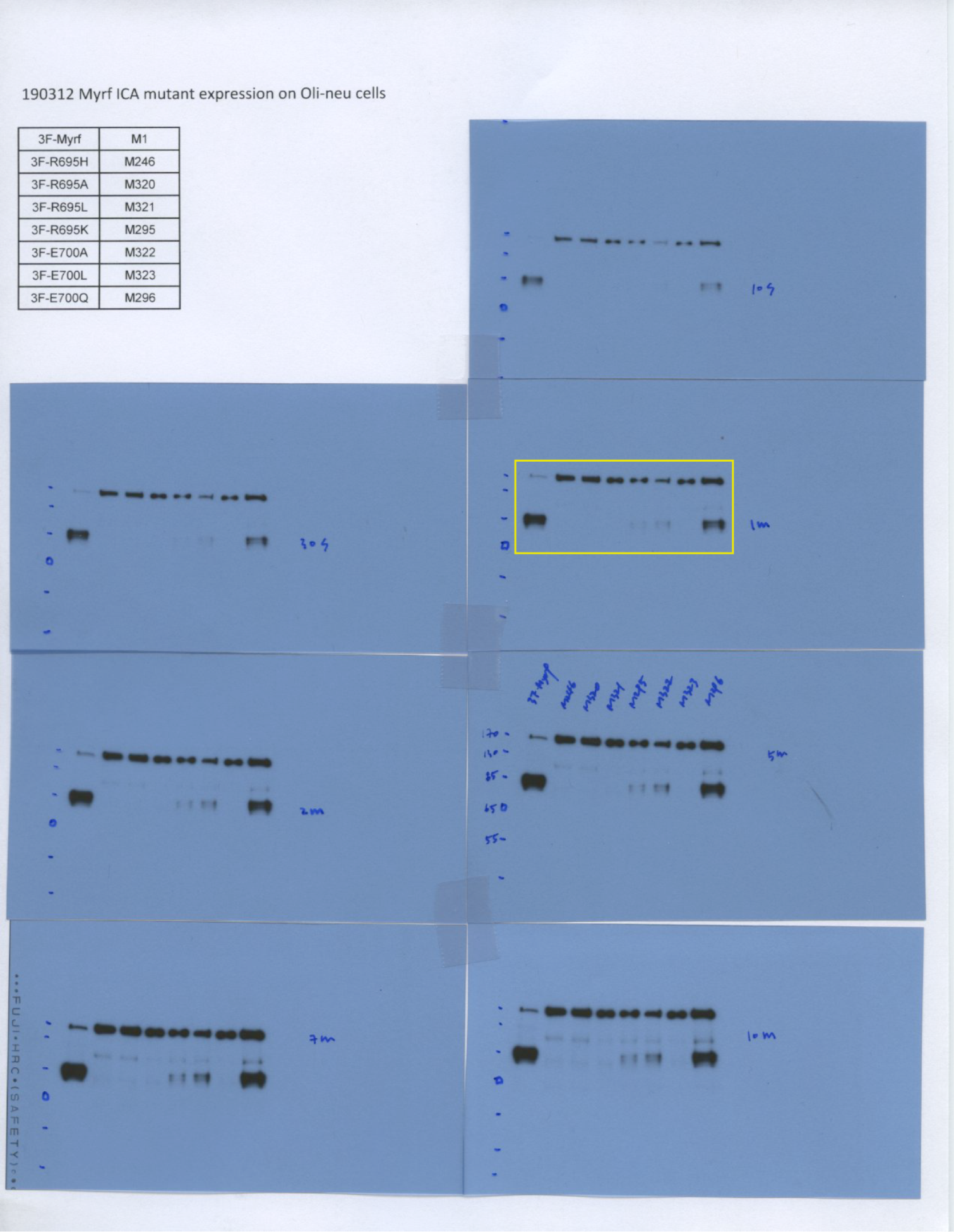
**

**Figure S8**

The raw Western blot results for Figure 5B in the main text. Portions marked by yellow boxes are the ones shown in Figure 5B.

**
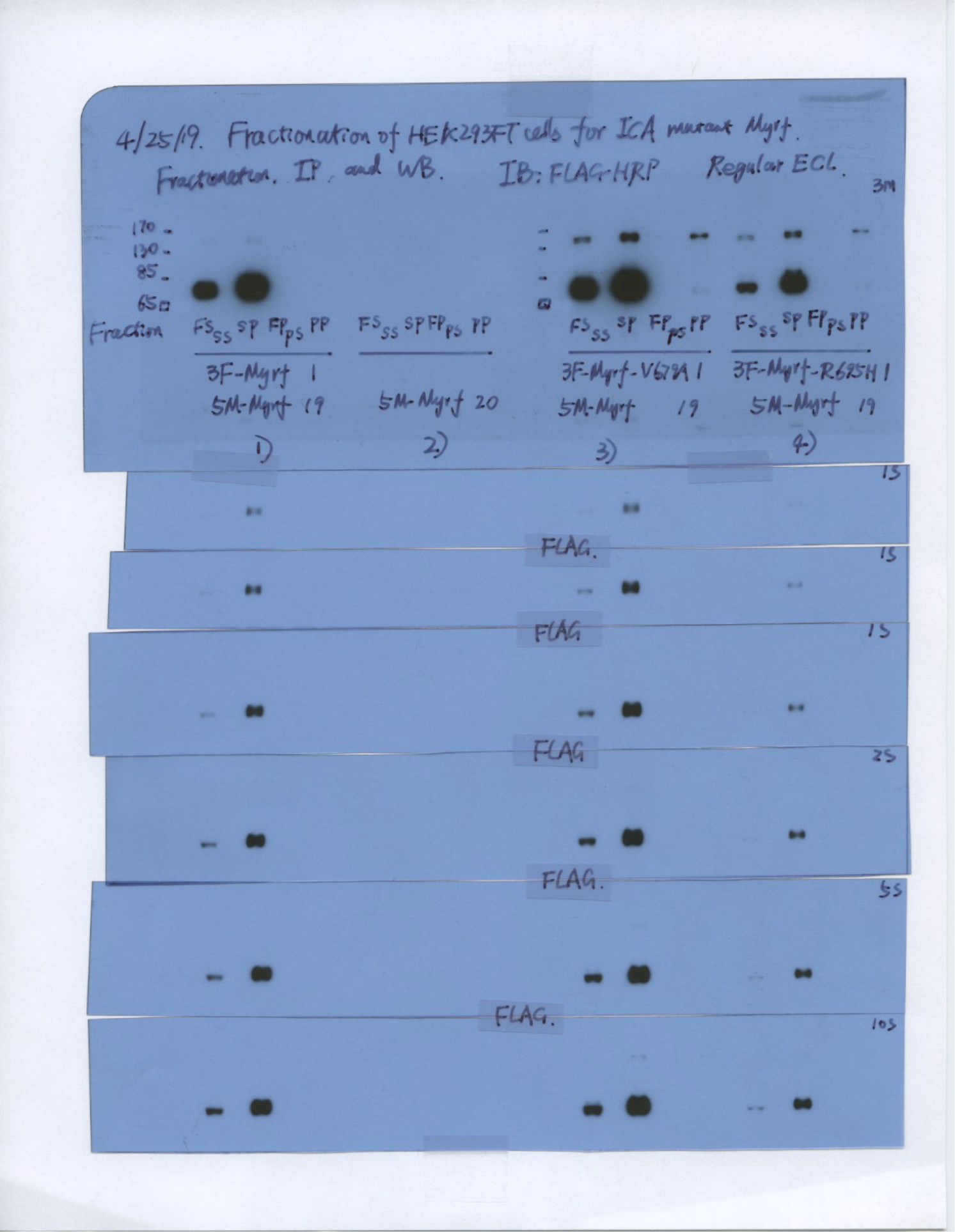
**

**
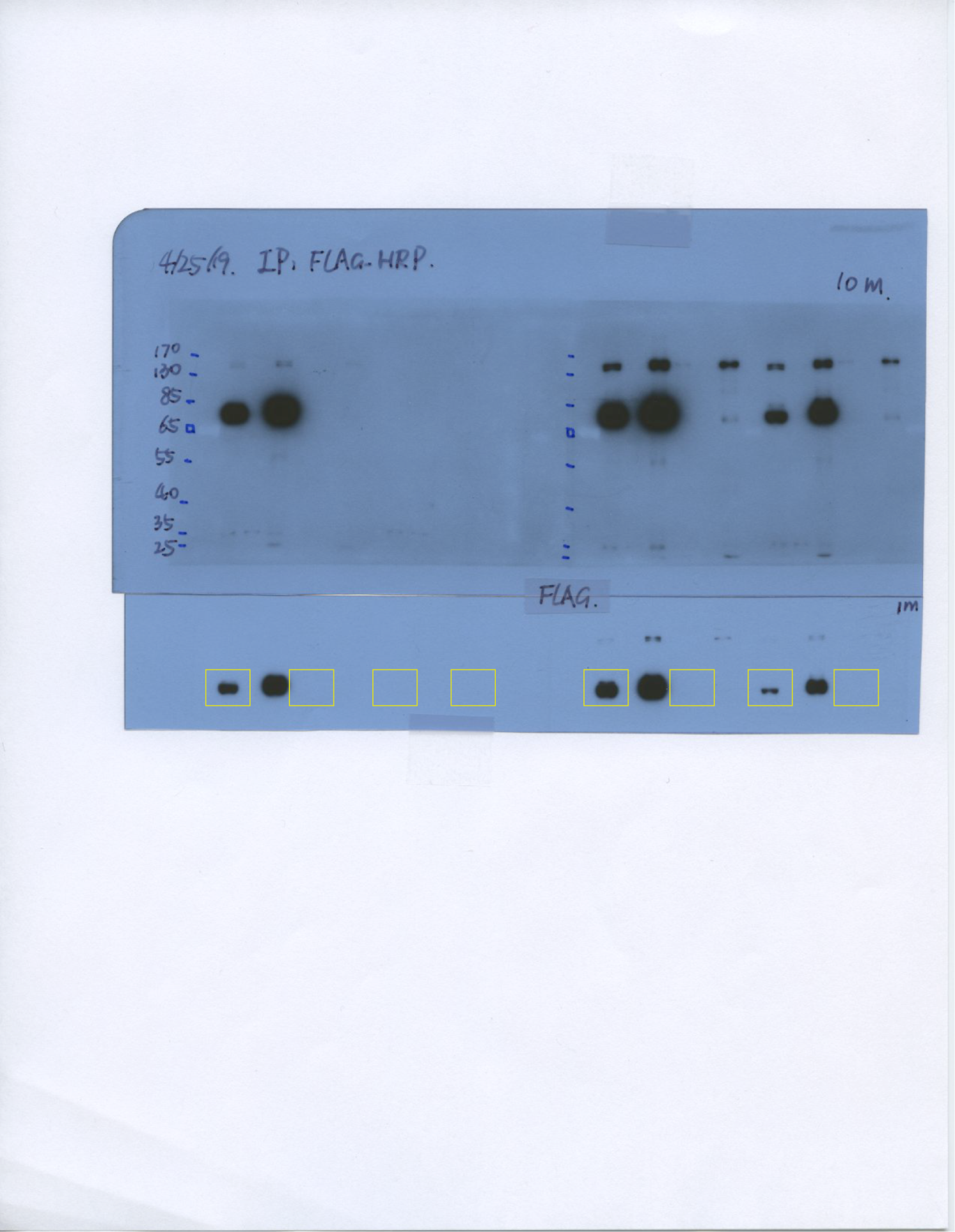
**

**Figure S9**

The raw Western blot results for Figure 5B in the main text. Portions marked by yellow boxes are the ones shown in Figure 5B.

**
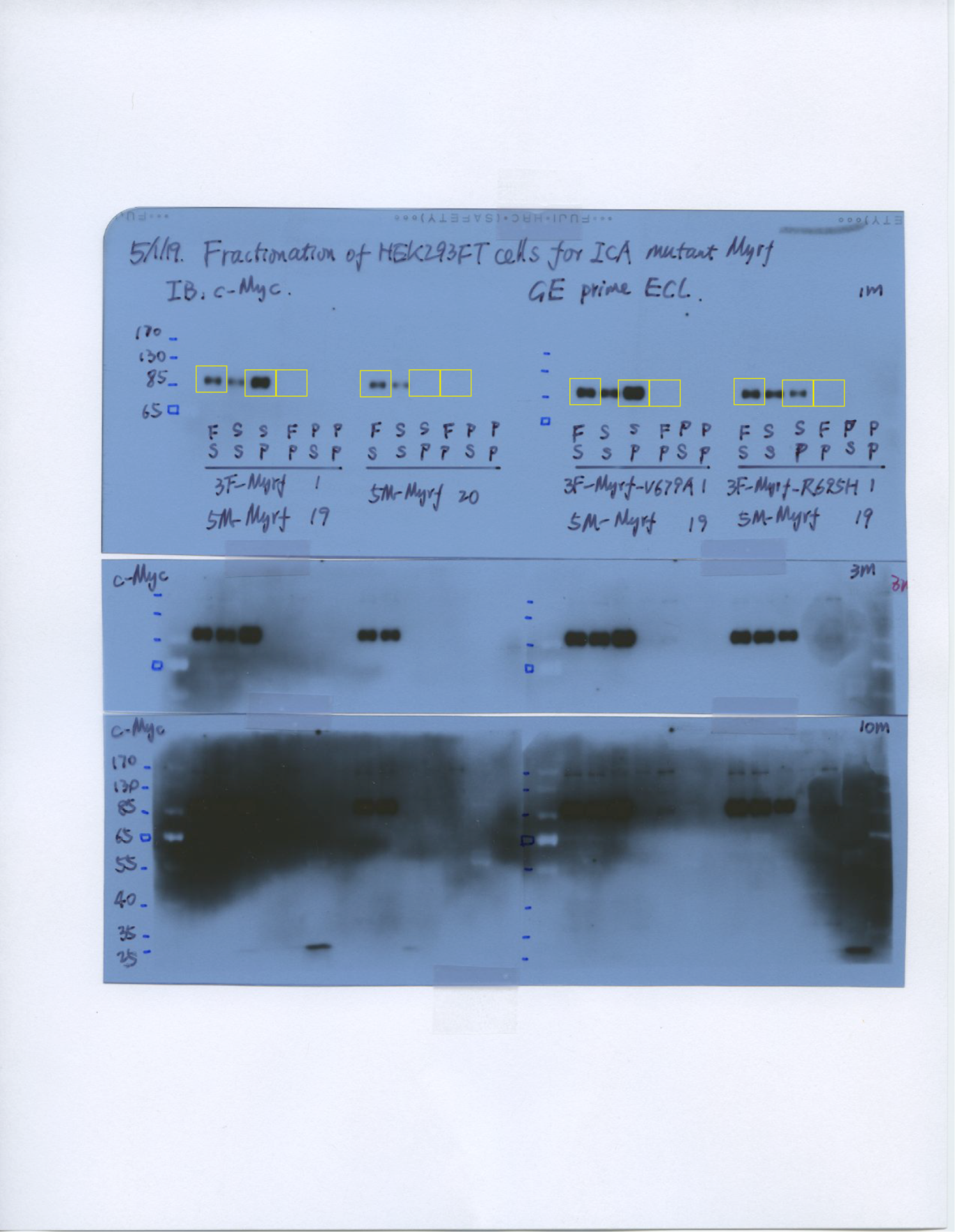
**

**Figure S10**

The raw Western blot results for Figure 5B in the main text. Portions marked by yellow boxes are the ones shown in Figure 5B.

**
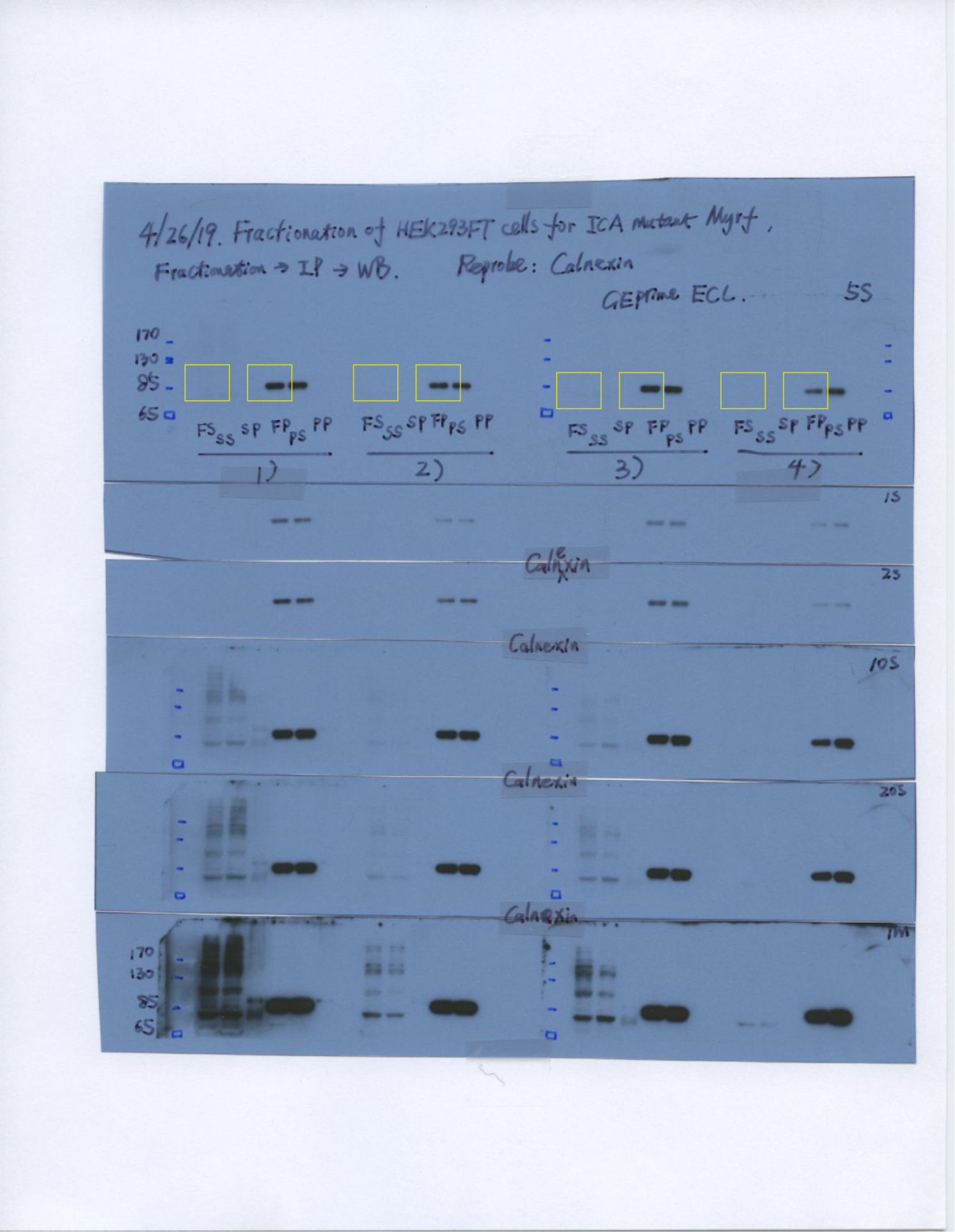
**

**Figure S11**

The raw Western blot results for Figure 5B in the main text. Portions marked by yellow boxes are the ones shown in Figure 5B.


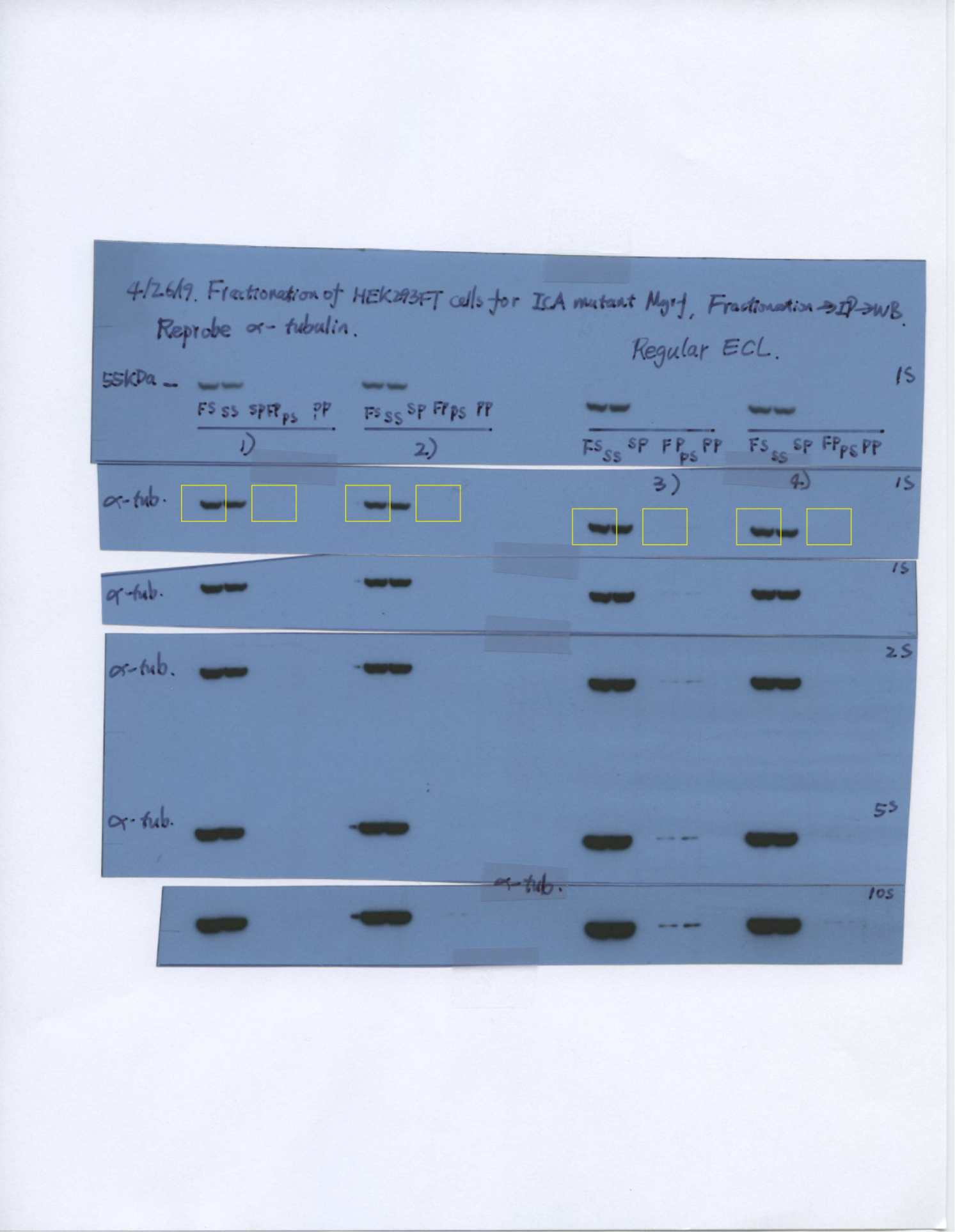


.
